# A beta-mixture quantile normalization method for correcting probe design bias in Illumina Infinium 450 k DNA methylation data

**DOI:** 10.1093/bioinformatics/bts680

**Published:** 2012-11-21

**Authors:** Andrew E. Teschendorff, Francesco Marabita, Matthias Lechner, Thomas Bartlett, Jesper Tegner, David Gomez-Cabrero, Stephan Beck

**Affiliations:** ^1^Statistical Genomics Group, UCL Cancer Institute, University College London, London WC1E 6BT, UK, ^2^Department of Medicine, Unit of Computational Medicine, Centre for Molecular Medicine, Karolinska Institute, Solna 171 76, Stockholm, Sweden and ^3^Medical Genomics Group, UCL Cancer Institute, University College London, London WC1E 6BT, UK

## Abstract

**Motivation:** The Illumina Infinium 450 k DNA Methylation Beadchip is a prime candidate technology for Epigenome-Wide Association Studies (EWAS). However, a difficulty associated with these beadarrays is that probes come in two different designs, characterized by widely different DNA methylation distributions and dynamic range, which may bias downstream analyses. A key statistical issue is therefore how best to adjust for the two different probe designs.

**Results:** Here we propose a novel model-based intra-array normalization strategy for 450 k data, called BMIQ (Beta MIxture Quantile dilation), to adjust the beta-values of type2 design probes into a statistical distribution characteristic of type1 probes. The strategy involves application of a three-state beta-mixture model to assign probes to methylation states, subsequent transformation of probabilities into quantiles and finally a methylation-dependent dilation transformation to preserve the monotonicity and continuity of the data. We validate our method on cell-line data, fresh frozen and paraffin-embedded tumour tissue samples and demonstrate that BMIQ compares favourably with two competing methods. Specifically, we show that BMIQ improves the robustness of the normalization procedure, reduces the technical variation and bias of type2 probe values and successfully eliminates the type1 enrichment bias caused by the lower dynamic range of type2 probes. BMIQ will be useful as a preprocessing step for any study using the Illumina Infinium 450 k platform.

**Availability:** BMIQ is freely available from http://code.google.com/p/bmiq/.

**Contact:** a.teschendorff@ucl.ac.uk

**Supplementary information:** Supplementary data are available at *Bioinformatics* online

## 1 INTRODUCTION

In the past few years, the field of epigenomics has risen to prominence (Feinberg, 2010; Petronis, 2010). Epigenomics not only offers an improved understanding of fundamental biological processes such as cellular differentiation and early embryogenesis, but is also widely recognized to be key in understanding the pathogenesis of complex genetic diseases like cancer (Baylin and Ohm, 2006; Feinberg *et al.*, 2006; Jones and Baylin, 2007). One particular epigenetic mark of interest is DNA methylation. Indeed, DNA methylation markers have been proposed as early detection, diagnostic and prognostic markers in a wide range of different diseases (Rakyan *et al.*, 2011). Underpinning this increased interest in epigenomics are significant advances in beadarray technology, which now allow routine measurement of DNA methylation at over thousands of CpG dinucleotides (Bibikova *et al.*, 2009, 2011; Sandoval *et al.*, 2011). Among these, the Illumina Infinium 450 k Human Methylation Beadchip offers both scalability and coverage (>480 000 probes) and is thus suitable for Epigenome-Wide Association Studies (EWAS) (Dedeurwaerder *et al.*, 2011; Rakyan *et al.*, 2011; Sandoval *et al.*, 2011).

A key statistical issue with the Illumina 450 k beadchip is that probes come in two different designs, which causes the methylation values derived from these two designs to exhibit widely different distributions (Dedeurwaerder *et al.*, 2011). Indeed, type2 probes are typically characterized by a much lower dynamic range compared with type1 probes, even after adjustment for differences in biological characteristics such as CpG density (Dedeurwaerder *et al.*, 2011). Comparison with bisulphite pyrosequencing data further showed that type2 probe values are biased and generally less reproducible (Dedeurwaerder *et al.*, 2011). To correct for this bias, a peak-based correction (PBC) method was proposed (Dedeurwaerder *et al.*, 2011) which normalises type2 design probes so as to render them comparable with type1 probes. Making the statistical distributions of type1 and type2 probes comparable is important for several reasons. Not doing so may introduce an enrichment bias towards type1 probes when ranking probes in supervised analyses, as the dynamic range of type1 probes is significantly higher. Moreover, methods that seek to determine differentially methylated regions (Jaffe *et al.*, 2012) also assume that probes within these regions are comparable and thus one would want to avoid any sources of technical variation within them. Finally, one would wish to apply unsupervised dimensional reduction algorithms (Houseman *et al.*, 2008; Koestler *et al.*, 2010) and classification algorithms (Zhuang *et al.*, 2012) to one single dataset, and not separately to two different assays.

Although the PBC method was validated in one dataset (Dedeurwaerder *et al.*, 2011) and has now been implemented in a pipeline for 450 k data (Wang *et al.*, 2012), two recent studies have exposed potential problems with PBC, specially when applied to tissue samples (Maksimovic *et al.*, 2012; Touleimat and Tost, 2012). In fact, as noted in these studies, PBC breaks down when the methylation density distribution does not exhibit well-defined peaks/modes. Hence, both studies proposed subset quantile normalization methods (SQN and SWAN) to correct for the type2 bias and which avoid the pitfalls of PBC (Maksimovic *et al.*, 2012; Touleimat and Tost, 2012). In this work, we show that PBC often leads to discontinuities (‘holes’) in the type2 density distribution. To address this problem, we here propose a novel mixture model-based normalization algorithm, called Beta MIxture Quantile dilation (BMIQ). We subject BMIQ to a rigorous evaluation using numerous independent datasets and using a number of different evaluation criteria to assess its robustness and performance. Specifically, we assess BMIQ in terms of reducing (i) the technical variance, (ii) the type2 bias, (iii) and the above-mentioned type1 enrichment bias. We further benchmark BMIQ against PBC and SWAN. For assessing technical variance and to allow a comprehensive comparison of BMIQ to PBC/SWAN across many datasets, we use in addition to replicates, a novel evaluation framework based on using adjacent type1–type2 probe pairs within probe clusters, a framework which we show leads to consistent and robust conclusions across 10 independent datasets. We demonstrate that, overall, BMIQ compares favourably to PBC and SWAN.

## 2 METHODS

### 2.1 Biological data: DNA methylation

#### Illumina Infinium 450 k DNAm assay

The DNA methylation data considered in this work were all generated using Illumina’s Infinium Human Methylation 450 k beadchip. Full details of this technology are described in Bibikova *et al.* (2011) and Sandoval *et al.* (2011). Briefly, the methylation value of each probe follows an approximate 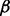-valued distribution, with 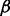 constrained to lie between 0 (unmethylated locus) and 1 (methylated). This follows from the definition of 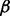 as the ratio of methylated to combined intensity values, i.e
(1)
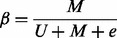

where 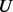 and 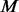 are the unmethylated and methylated intensity values of the probe (averaged over bead replicates) and 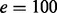 is a small correction term to regularize probes of low total signal intensity (i.e. probes with 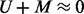 after background subtraction). Throughout we used non–background-corrected DNAm data. Of the 485 577 probes, 72% are of a type2 design in which the 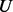 and 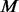 measurements are obtained in different colour channels, while the rest (28%) of the probes are of the old type1 design in which both 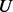 and 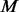 measurements are obtained in the same colour channel. Importantly, type1 and type2 probes differ significantly in terms of CpG density, with CpGs mapping to CpGs islands overrepresented among type1 probes (Bibikova *et al.*, 2011; Sandoval *et al.*, 2011).

#### Datasets 1 and 2: (BT) and (CL)

This is a subset of the 450 k dataset considered in Dedeurwaerder *et al.* (2011). We used the data from eight fresh frozen (FF) breast tumours and eight normal breast tissue specimens [hereafter refered to as (BT)], as well as the three replicates from the HCT116 WT cell-line [hereafer refered to as (CL)]. For these cell-lines, matched bisulphite pyrosequencing (BPS) data were available for nine type2 probes.

#### Datasets 3 and 4: (FFPE) and (FF)

This 450 k dataset consists of 32 formalin-fixed paraffin-embedded (FFPE) head and neck cancers (HNCs), of which 18 were HPV+ and 14 HPV−, as well as five fresh frozen HNCs (FF), of which 2 were HPV+ and 3 HPV−. The data are available from GEO under accession number GSE38271.

#### Dataset 5: (GBM)

This 450 k dataset consists of 81 glioblastoma multiformes (GBMs) (Turcan *et al.*, 2012), 49 of which were categorized as CpG island methylator positive (CIMP+) and 32 as CIMP−.

#### Datasets 6–10: TCGA, LIV, LC, BLDC, HCC

These 450 k samples are all from the TCGA: Specifically, Dataset6 (TCGA) consists of 10 samples as provided in the Bioconductor data package TCGAmethylation 450 k, Dataset7 (LIV) consists of nine normal liver tissue samples from Batch203 in the TCGA data portal, Dataset8 (LC) consists of 22 lung cancer samples from Batch196, Dataset9 (BLDC) consists of 12 bladder cancer samples from Batch86 and Dataset10 (HCC) consists of 10 hepatocellular carcinoma samples from Batch153.

### 2.2 BMIQ: Beta MIxture Quantile dilation normalization strategy

The normalization of type2 probe values into type1 must satisfy the following criteria. (i) It must allow for the different biological characteristics of type1 and type2 probes, i.e type1 probes are significantly more likely to map to CpG islands than type2 probes, and hence the relative proportion of methylated and unmethylated probes will vary between the two designs. In the case of the type2 probes, this means that these proportions must be invariant under the normalization transformation. (ii) The transformation of the type2 probe values should reduce the bias, which amounts to matching of the density distributions of the two design types, specially at the unmethylated and methylated extremes. (iii) The transformation must be monotonic, that is, the relative ranking of beta values of the type2 probes must be invariant under the transformation. Next, we propose a normalization strategy for the type2 probes satisfying the above properties and which is based on three steps:

- Fitting of a three-state (unmethylated-U, hemimethylated-H, fully methylated-M) beta mixture model to the type1 and type2 probes separately. For sake of convenience we refer to intermediate allelic methylation as hemimethylation even though hemimethylation is most often used in the context of strand-specific methylation. Let 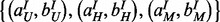 denote the parameters of the three beta distributions for the type1 probes, and similarly let 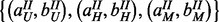 describe the estimated parameters of the three beta components for the type2 probes. State membership of individual probes is determined by the maximum probability criterion.
- For those type2 probes assigned to the U-state, transform their probabilities of belonging to the U-state to quantiles using the inverse of the cumulative beta distribution with beta parameters 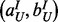 estimated from the type1 U component. Let 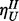 denote the normalized values of the type2 U-probes.
- For those type2 probes assigned to the M-state, transform their probabilities of belonging to the M-state to quantiles using the inverse of the cumulative beta distribution with beta parameters 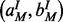 estimated from the type1 M component. Let 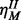 denote the normalized values of the type2 M-probes.
- For the type2 probes assigned to the H-state, we perform a dilation (scale) transformation to ‘fit’ the data into the ‘gap’ with endpoints defined by 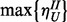 and 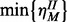.

We next describe each of the above steps in detail. We first model each beta value 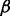 as,
(2)
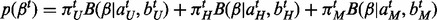

where 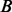 denotes the beta probability density function and 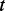 denotes the design type 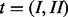. We infer the parameters 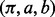 using an Expectation Maximization (EM) algorithm as described in (Ji *et al.*, 2005). The estimated parameters we denote again by 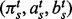 where 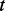 labels the design and 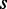 one of the three states (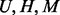). The resulting means of the estimated beta-distributions are denoted by 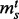 where
(3)
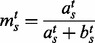



Further, let 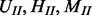 denote the set of type2 probes assigned to unmethylated, hemimethylated or fully methylated states (using the maximum probability criterion), and let 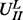 (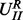) denote the set of 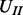 probes with 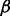-values smaller (larger) than 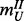. Similarly, let 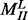 (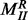) denote the set of 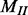 probes with 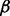-values smaller (larger) than 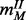. This subdivision into values which fall left (L) or right (R) of the mean are necessary since the state membership probabilities estimated from the EM algorithm are two tailed. Next, for the 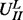 probes we estimate their type2 tail probabilities of belonging to the 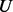-state, i.e 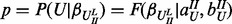 where 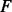 denotes the cumulative distribution beta function. We then transform these probabilities back to quantiles (i.e 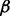-values), but using the type1 parameters, i.e
(4)
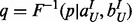

and finally set the normalized 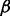-value, 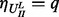. An identical transformation (using 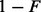 instead of 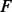) is performed for the 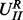 probes. Next, we perform the analogous operation for the 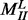 and 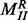 probes. This therefore yields normalized type2 values for all type2 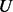 and 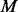 probes.

Finally, it remains to normalize the type2 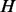 probes. Since the type2 H probe value distribution is sandwiched between the U and M probe distributions, we can use an empirical approach to normalize these values, thus also bypassing the difficulty that type2 H probe values are not well described by a beta distribution (Supplementary Fig. S1). Specifically, we first identify the minima and maxima of the type2 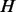-probes, 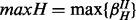 and 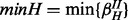, and let 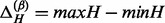. We also find the minimum of the 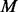-probes, i.e 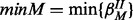 and the maximum of the 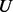-probes, i.e. 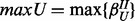. We point out that in fact all of these extrema represent robust values, because they do not represent extrema on the bounded (0,1) support, i.e. the values 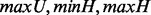 and 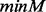 are not close to 0 or 1. Next, we define distances

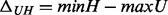


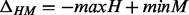



We want the new normalized maximum and minimum values of 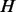-probes to satisfy

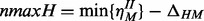


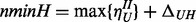

so that 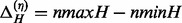. The normalized 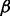-values for the 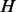-probes is then given by the conformal (shift + dilation) transformation
(5)
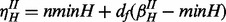

where 
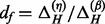
 is the dilation factor. It is important to observe that the conformal transformation involves a non-uniform rescaling of the H probe beta values since it depends on the beta-value of the probe. This is absolutely key in order to avoid gaps or holes from emerging in the normalized distribution.

This algorithm is flexible in that the dilation can be performed also including the 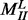 (and/or the 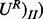 probes, which means that the matching of the density distributions is only done on the respective tails (i.e. the 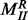 and 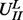 probes). We point that in practice we find that the optimal performance is attained by including the 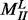 probes with the 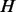-probes when performing the conformal transformation. This is because we observed that it is the left tail end of the methylated type2 distribution that is generally not well described by a beta-distribution (Supplementary Fig. S1), presumably as a result of the dye bias, which is specific to the type2 distribution.

There are a number of other important points to note about BMIQ: (i) First, it is important to choose reasonable initial weight parameters 
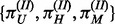
 in the EM-algorithm. As these fractions can vary significantly from study to study, or even sample to sample depending on the nature of the samples assayed, it is important to choose reasonable initial values on a per-sample basis. Not doing so may result in mild discontinuities in the type2 density distribution. To obtain estimates for these prior weight parameters, we first note that their estimation only requires estimates for the two thresholds used for calling the three states, since the weights for a given sample are determined given a choice of thresholds. Moreover, although the thresholds will show little inter-sample variability, the weights may not, reflecting the biological differences in the number of probes that are unmethylated, hemimethylated or fully methylated. In BMIQ, the estimation of the initial thresholds proceeds in an automatic fashion on a per-sample basis: in detail, we use the estimated thresholds from the type1 distribution (which always gives an excellent fit, Supplementary Fig. S1) to then obtain type2-specific thresholds using a simple correction reflecting the difference in the modes between the type1 and type2 distributions. Specifically, if 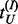 is the lower threshold (i.e. type1 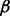 values less than 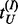 are called unmethylated) and 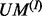 and 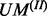 are the estimated modes of the unmethylated type1 and type2 components, the intial prior estimate for 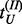 would be 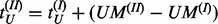. Similarly, the threshold for calling probes fully methylated or just hemi-methylated would be 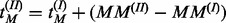 where 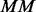 denotes the mode of the methylated state. We note that resulting thresholds would normally fall within the ranges 0.2–0.3 and 0.60–0.8, respectively. Having thus identified reasonable initial estimates for the weights 
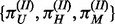
, the algorithm will then automatically determine the unmethylated, hemimethylated and methylated fractions for each sample individually. (ii) A second important observation is the overall robustness of BMIQ to the goodness of the type2 EM-fit. This is important, since we consistenly observe that the methylated type2 distribution is not well described by a beta function (Supplementary Fig. S1). In this regard we have also verified that using a beta mixture model with more than three states does not improve the overall type2 fit. Fortunately however, as explained above, the goodness of fit problem associated with the 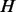 and 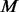-probes can be easily circumvented by modelling only the right tail of the methylated component as the corresponding tail of a beta distribution. In this case, the left tail is modelled together with the 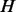-probes using the observed *empirical* distribution. Hence, the probe values that are not well described by a beta distribution are *not* normalized using estimated beta parameters, which means that their normalization is insensitive to the goodness of fit.

## 3 RESULTS

### 3.1 Improved robustness of BMIQ

**Fig. 1.**
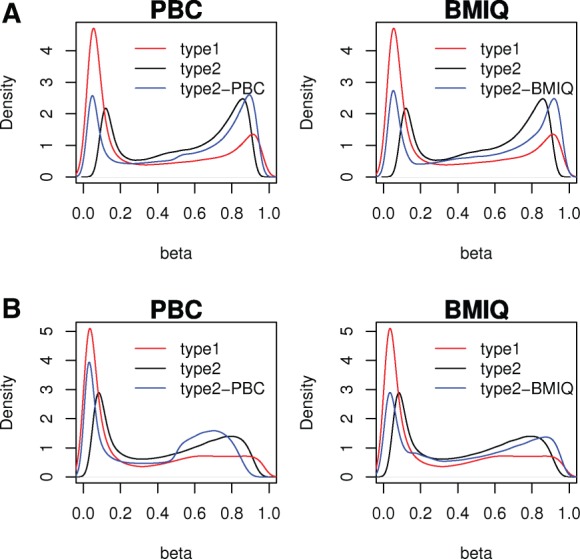
(**A**) Density distributions of beta-values for the type1 probes, type2 probes (unnormalized) and normalized type2 probes for a breast tumour sample from Dataset1. (**B**) Density distributions of beta-values for the type1 probes, type2 probes (unnormalized) and normalized type2 probes for a head and neck tumour sample from Dataset3. Left panels are for PBC, right panels for BMIQ

To validate BMIQ, we first applied it to data where the PBC method has been shown to work reasonably well. Thus, we applied it to a fresh frozen breast tumour sample from Dataset1 (Dedeurwaerder *et al.*, 2011) (Fig. 1A). We can see that for this particular sample, the methylated type1 peak is well defined and as a result both PBC and BMIQ appear to do well in generating smooth density distributions for the type2 probes, which at the methylation extremes are also reasonably well matched to the type1 density distribution. Next, we applied both PBC and BMIQ to the FFPE tumour samples from Dataset3, for which the type1 methylated peak was not well defined (Fig. 1B). In these samples, PBC generated a type2 density distribution that exhibited relatively sharp changes (‘holes’) (Fig. 1B), suggestive of a non-optimal adjustment and indicating that in such cases PBC breaks down. This is not suprising since PBC relies heavily on the ability to detect clear unmethylated and methylated modes in the type1 density distribution in order to then adjust the type2 distribution accordingly. Importantly, BMIQ does not use the type1 modes to adjust the type2 data, and hence BMIQ normalization of the type2 probes generated a much smoother density distribution, suggestive of an improved normalization framework (Fig. 1B). Moreover, the tail ends of the BMIQ type2 distribution better matched those of the type1 distribution without affecting the fractions of unmethylated, hemimethylated and fully methylated probes, which are preserved by the BMIQ transformation.

### 3.2 BMIQ reduces technical variation

**Fig. 2.**
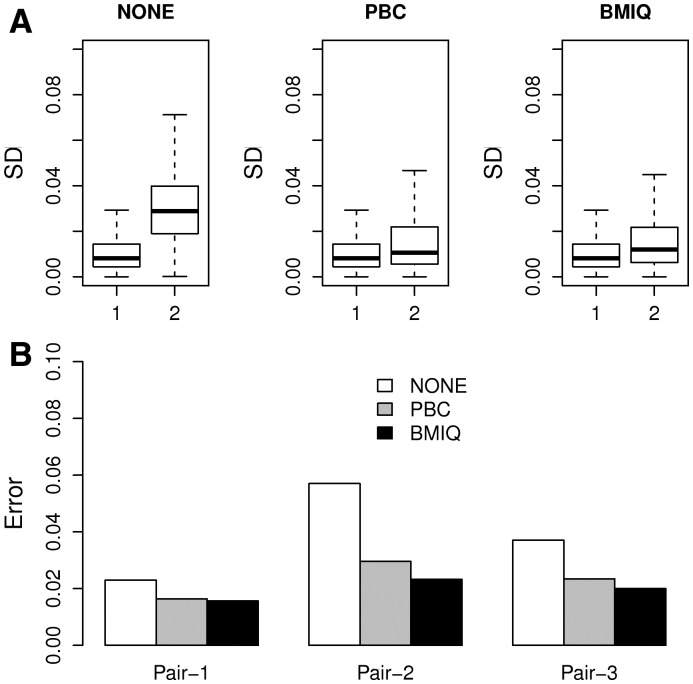
(**A**) Boxplots comparing the standard deviation (*y*-axis) of type1 (1) and type2 (2) probes over the three HCT116 WT replicates from (Dedeurwaerder *et al.*, 2011), for the case of no design normalization (NONE), PBC and BMIQ. (**B**) As (A) but now comparing the Manhattan distances over type2 probes only for each pair of replicates

To further test BMIQ we applied it to Dataset2 (CL) consisting of three replicates of a given cell-line, to investigate if reproducibility is improved. First, we computed for each of the probes its standard deviation across the three replicates and for each of the three scenarios: no normalization, PBC and BMIQ. As seen, BMIQ performed similarly to PBC and led to a significant reduction in inter-replicate variability (Fig. 2A). To check this further, we compared the normalization methods in terms of the Euclidean distance between the three possible pairs of replicates across the type2 probes (Fig. 2B). Using this measure, BMIQ not only led to a significant improvement, but was also marginally better than PBC (Fig. 2B).

### 3.3 BMIQ reduces bias of type2 methylation values

**Fig. 3.**
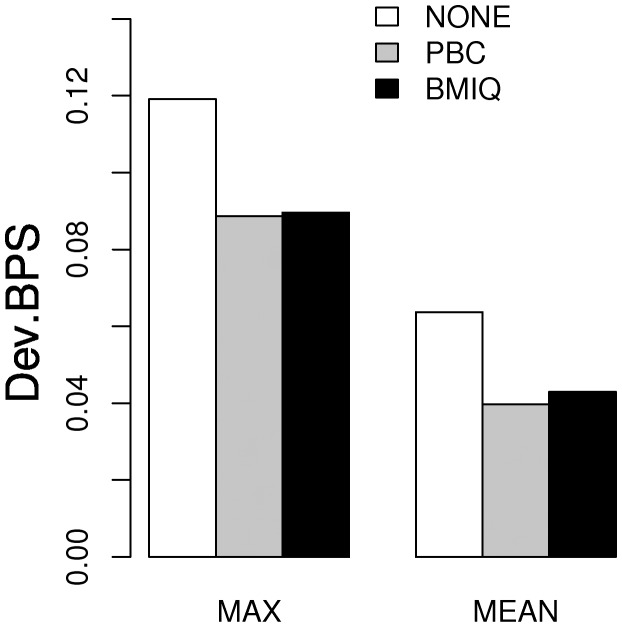
Barplot comparing the maximum and mean absolute deviation of 450 k values from their corresponding bisulphite pyrosquencing values over the nine type2 probes considered in Dedeurwaerder *et al.*, (2011). We compare these deviation measures for the case of no design normalization (NONE), PBC normalization and BMIQ

Using replicates to evaluate normalization methods assesses the method in terms of reducing technical variability but does not evaluate whether the actual values of the replicates are closer to the true estimate. This requires comparison with a gold-standard, which is provided by matched BPS data (Dedeurwaerder *et al.*, 2011). Hence, we compared the methods in terms of the deviations from BPS methylation values for the nine type2 probes in Dedeurwaerder *et al.* (2011) for which matched 450 k BPS data were available. Similar to PBC, we observed that BMIQ significantly reduced the bias of type2 values (Fig. 3), although there was no improvement over PBC itself, presumably owing to the fact that in these specific samples the methylated type1 peak was well defined, a scenario in which PBC works well.

### 3.4 BMIQ eliminates the type1 enrichment bias

**Fig. 4.**
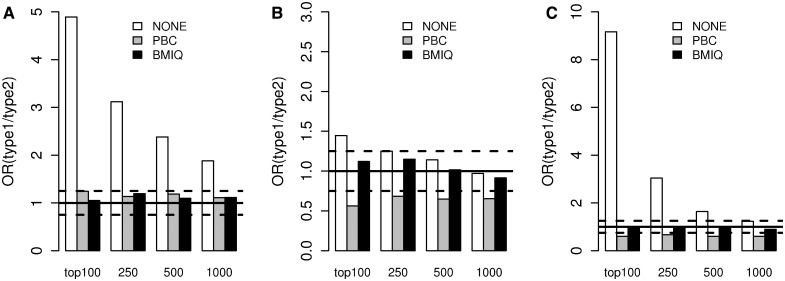
OR of relative enrichment of type1 probes over type2 probes among the top 100, top 250, top 500 and top 1000 ranked probes, where probes were ranked according to the absolute difference in methylation (beta-values). ORs are shown for the case of no design normalization (NONE), PBC and BMIQ normalization. Supervised analysis and ranking was performed only on probes mapping to CpG islands and within 200 bp upstream of transcription start site to correct for biological differences between type1 and type2 probes. The line OR = 1 represents the ideal scenario of no relative enrichment of type1 versus type2 probes. The 95% confidence envelope around OR = 1 is shown to assess significant deviations from OR = 1. (**A**) Eight breast cancers versus eight normal breast (Dataset1), (**B**) 18 HPV+ HNCs versus 14 HPV− HNCs (Dataset3), (**C**) 49 CIMP+ GBMs versus 32 CIMP− GBMs (Dataset5)

To further test BMIQ, we considered the supervised context, in which a ranked list of probes correlating with a phenotype of interest is derived. Given the higher dynamic range of type1 probes, one expects that this would favour type1 probes and that therefore there would be a relative over enrichment of type1 over type2 probes in a top ranked list of probes. However, one key difficulty when assessing whether there is a bias towards type1 probes is that type1 and type2 probes differ significantly in terms of their biological characteristics, in particular in terms of CpG density. Hence, in order to avoid confounding by CpG density, we only selected probes that mapped to CpG islands and to 200 bp upstream of the TSS, thus allowing a sensible comparison between type1 and type2 probes. We considered three different datasets and derived for each a ranked list of probes associated with a phenotype of interest: breast cancer versus normal breast [Dataset1 (BT)], HPV + versus HPV−HNC [Dataset3 (FFPE)], and CIMP+ versus CIMP− (GBM) (Dataset5). The ranking was performed using the magnitude of differential methylation. Although this ranking does not take the within-phenotype variability into account, it remains a popular method (Dedeurwaerder *et al.*, 2011; Du *et al.*, 2010), and for our purposes, using the absolute difference in beta-values allows us to better interpret the performance of the different normalization methods. To assess any potential bias towards type1 probes, we computed for a given number of top ranked probes the odds ratio (OR) of relative enrichment of type1 over type2 probes. Across all three datasets, we indeed observed a bias towards type1 probes, although the severity of this bias varied substantially from study to study (Fig. 4). Using PBC, in one dataset this bias was eliminated; however, in the other two datasets, PBC overcorrected the data leading to a bias favouring type2 probes. In contrast, BMIQ eliminated the type1 enrichment bias in all three datasets (the resulting OR was always close to 1) without overcorrecting the data and avoiding the type2 enrichment bias seen for PBC.

### 3.5 Reduced technical variability within probe clusters

**Fig. 5.**
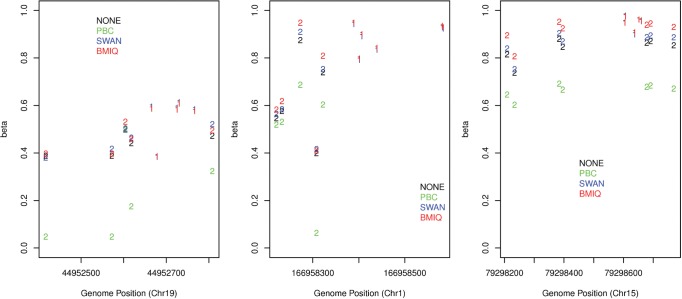
Examples of methylation profiles, from Dataset4(FF), of three probe clusters on chromosomes 19, 1 and 15, respectively. The design type of each probe is indicated with 1 and 2. The non design normalized data (NONE), PBC, SWAN and BMIQ corrected data are superimposed. Observe how across the three loci, BMIQ generally corrects the data in a way which is more consistent with the neighbouring type1 values. In the left panel PBC overcorrects the data, in the right panel there is suppression, while in the middle panel PBC both overcorrects and suppresses beta values. The data values can be found in Supplementary Table S1

To further assess BMIQ, we devised an evaluation framework which exploits the well known spatial correlation of DNA methylation at scales <500 bp (Eckhardt *et al.*, 2006). Approximately 27% of the 450 k probes fall into 12 501 probe clusters, defined as contiguous regions containing at least seven probes with no two adjacent probes separated by >300 bp (Jaffe *et al.*, 2012). Within these probe clusters, we posited that pairs of adjacent probes, one from each design and within 200 bp of each other, should have similar methylation values. Among the 12 501 probe clusters we identified on the order of ∼30 000 of such adjacent type1–type2 probe pairs. Thus, to evaluate the normalization algorithms, we asked which one minimizes the absolute difference in methylation between such closely adjacent type1–type2 pairs. We considered a total of 10 independent datasets, seven of which had idat files, thus allowing also for a direct comparison with SWAN (Maksimovic *et al.*, 2012). For each dataset, we computed the mean of the absolute deviations over probe pairs and samples. Comparison of these average deviations revealed that BMIQ consistently reduced the technical variation, while also outperforming PBC and SWAN (Table 1). In fact, in 9 of 10 datasets, BMIQ was substantially better as assessed using a paired Wilcoxon rank sum test over all probe pairs and samples (Table 1). Example methylation profiles within these probe clusters confirmed that BMIQ successfully reduces the technical variability, while PBC can break down either overcorrecting or suppressing the type2 data values, leading to substantial differences in methylation between neighbouring probes, even at scales of <100 bp (Fig. 5 and Supplementary Table S1).

**Table 1.** For each dataset we compare the absolute deviation in methylation between adjacent type1–type2 probe pairs (probes within 200 bp of each other), averaged over probe pairs and samples, for four different normalisation methods

Dataset	NONE (%)	PBC (%)	SWAN (%)	BMIQ (%)	*P*
BT	7.8	6.3	NA	**6.2**	<10^−10^
CL	8.6	18.4	NA	**7.2**	<10^−10^
FFPE	9.2	8.0	8.5	**7.8**	<10^−10^
FF	8.5	8.1	7.6	**7.3**	<10^−10^
GBM	9.2	7.6	NA	**7.5**	<10^−10^
TCGA	9.4	7.8	8.3	**7.4**	<10^−10^
LIV	10.0	**6.3**	7.4	6.4	∼1
LC	10.3	7.0	7.7	**6.7**	<10^−10^
BLDC	11.0	8.0	7.9	**7.6**	<10^−10^
HCC	12.0	8.5	8.7	**8.1**	<10^−10^

NONE refers to the case of no adjustment for probe design type. The last column give the paired Wilcoxon rank sum test *P*-value (treating each probe-pair deviation in each sample as a separate value), assessing the statistical significance that the absolute deviation for BMIQ is smaller than the next best competing method. NA indicates non-available owing to lack of access to idat files needed for processing by SWAN. In bold-face we show the smallest deviation across methods.

### 3.6 BMIQ robustly identifies features associated with HPV status

Finally, it must be verified that the reduction in technical variance obtained with BMIQ is not at the expense of a reduced biological signal. Since it is difficult to establish what constitutes a true positive, we used a training test set strategy, to identify features in a training set and calling them true positives if validated in a test set. This strategy thus allows for a comparison of sensitivity and positive predictive value (PPV) between the different normalization methods. To perform this analysis, we used Dataset4(FF) consisting of 2 HPV+ and 3 HPV− fresh frozen head and neck cancers to derive features associated with HPV status. As test set we used Dataset3(FFPE) consisting of 18 HPV+ and 14 HPV− head and neck cancers (FFPE tissue). Using limma (Smyth, 2005) and an FDR threshold of 0.35, we observed that BMIQ identified substantially more differentially methylated features than PBC or SWAN (Table 2). Importantly, this was not at the expense of a smaller PPV, and so, overall, BMIQ identified substantially more true positives (Table 2).

**Table 2.** Table listing the number of differentially methylated probes (nDMPs) associated with HPV status in Dataset4 (FF), and the corresponding estimates for the positive predictive value (PPV) and number of true positives (nTPs) estimated using Dataset3 (FFPE) as test set

Metric	NONE	PBC	SWAN	BMIQ
nDMP	51	70	41	252
PPV	0.25	0.18	0.19	0.20
nTP	13	13	8	51

DMPs were defined at an FDR threshold of 0.35, and those with the same sign of limma *t*-statistic in the two sets and with a corresponding *P*-value < 0.01 in the test set were deemed true positives.

## 4 DISCUSSION

In this work we have presented a novel mixture-model-based algorithm (BMIQ) for correcting the bias associated with type2 probe values in 450 k studies. Confirming the observations made in Touleimat and Tost (2012) and Maksimovic *et al.* (2012), we have seen that PBC can break down in samples with ill-defined type1 methylation peaks, causing sharp, almost discontinuous changes (which we call ‘holes’) in the density distributions (Fig. 1B), which motivated our quest to find a more robust algorithm. We have shown that BMIQ improves the robustness and can successfully normalize the type2 distribution, avoiding the appearance of such ‘holes’ (Fig. 1B). Moreover, BMIQ successfully matches the tail-ends of the type1 and type2 distributions, while faithfully preserving the proportions of unmethylated and methylated probes within each of the two designs.

To further test BMIQ, we used data on technical replicates (to show that it reduces technical variability) and matched BPS data (to show that it reduces the bias of type2 probe values). Using these criteria, we have seen that BMIQ leads to significant improvements, similar to the improvements noted for PBC (Figs 2 and 3). In relation to these evaluation criteria, it is worth pointing out that BMIQ was compared with PBC on samples with well-defined type1 methylation peaks, i.e. on data that were used to develop PBC itself. Hence, it is likely that an evaluation of technical reproducibility (using replicates) and type2 value bias (using matched BPS data) on data where the methylated type1 peaks are less well-defined would favour BMIQ over PBC. However, we did not have access to technical replicates or matched BPS data in the other specific datasets considered here. Therefore, in order to further assess BMIQ, we devised a supervised framework across three independent datasets to objectively compare the algorithms in their ability to reduce the expected enrichment bias of type1 probes. First, we showed that if no design normalization is performed then there is indeed an enrichment bias towards type1 probes, even when adjusted for CpG density (Fig. 4). We also showed that in two datasets, PBC overcorrected the type2 data, leading to an overinflated dynamic range, thus favouring type2 probes and causing an ‘overshooting’ of the enrichment scores, reflected by a significant underenrichment of type1 probes (Fig. 4B and C). In contrast, BMIQ successfully avoided any type1/type2 enrichment bias in all three datasets, indicative of an improved normalization of type2 values (Fig. 4). We should point out that the overcorrection of type2 values and the associated overinflated dynamic range caused by PBC is consistent with the presence of ‘holes’ in the hemimethylated region of the type2 density distribution. Thus, with PBC there is an artificial expulsion of data points from the hemimethylated region to the unmethylated and/or methylated extremes. In a further assessment of BMIQ, we conducted a detailed spatial analysis of DNA methylation at the level of probe clusters across 10 independent datasets. By carefully analysing adjacent type1–type2 probe pairs, we observed that PBC can often overcorrect or suppress the data (in some cases inducing abnormally large 30% changes in methylation), in contrast to BMIQ, which normalized type2 values in a way that rendered them more consistent with the values of neighbouring type1 probes (Fig. 5 and Table 1). Interestingly, BMIQ also appeared to outperform SWAN (Table 1), which is part of the popular and widely used *minfi* package (Hansen and Aryee, 2012). Of note, the reduction in technical variance achieved by BMIQ was not at the expense of a lower biological signal (Table 2).

In summary, using a number of different evaluation criteria and numerous datasets, we have seen that BMIQ compares favourably with both PBC and SWAN. Although we did not compare BMIQ to SQN (Touleimat and Tost, 2012), this latter method is very similar to SWAN, as they both rely on a probe subset quantile normalization. Like SQN/SWAN, BMIQ uses quantiles to normalize the type2 probe values into a distribution that is comparable with that of type1 probes. However, unlike SQN and SWAN, BMIQ is based on an explicit beta-mixture modeling framework, and uses state-membership probabilities under this beta mixture model to reassign the quantiles of the type2 probes according to the type1 distribution. Thus, BMIQ is assumption-free, as it does not require a separate normalization to be performed on selected subsets of probes that are matched for biological characteristics (e.g. CpG density), as done in SQN and SWAN. In fact, under the BMIQ framework, all the biological differences (including CpG density) between the type1 and type2 probes are captured by the estimated fractions of unmethylated, hemimethylated and methylated probes, which will be different between the two assays. Thus, BMIQ does not depend on a priori and somewhat arbitrary choices of which biological characteristics to use when matching the type1 and type2 distributions. For instance, in SQN the normalization is performed on probe subsets defined by specific CpG characteristics (e.g. shelves, shores, CpG islands); however, multiple different definitions for say CpG islands exist (Gardiner-Garden and Frommer, 1987; Takai and Jones, 2002; Wu *et al.*, 2010; Zhao *et al.*, 2009). Similarly, in SWAN the number of CpGs in the probe body, even if they differ by one, is used to define probe normalization categories, and thus it is unclear whether these probe categories represent an optimal way of dividing the probes up. Therefore, we see the beta-mixture model framework of BMIQ as an important conceptual advantage over SQN/SWAN, since, as demonstrated here, it successfully normalises type2 probe values, faithfully preserving the numerous and complex biological differences that exist between the two designs without ever needing to define probe subsets. Nevertheless, it will be interesting to conduct a comprehensive and detailed comparison of BMIQ, SQN and SWAN on matched 450 k BPS data on a sufficiently large number of loci and samples.

## 5 CONCLUSIONS

We have presented a mixture model assumption-free normalization algorithm, BMIQ, which will be useful for correcting the bias associated with the type2 assay in DNA methylation studies using the Illumina Infinium 450 k platform.

*Funding*: A.E.T. is supported by a Heller Research Fellowship. M.L. was supported by a Wellcome Trust Research Training Fellowship (093855). S.B. was supported by a Royal Society Wolfson Research Merit Award (WM100023) and grants from the Wellcome Trust (084071) and EU-FP7 BLUEPRINT (282510). We also thank FP7 SYNERGY-COPD (F.B., D.G., J.T.), BILS (D.G.) and Stockholm County (J.T.).

*Conflict of Interest:* none declared.
